# Dexmedetomidine Protects against Airway Inflammation and Airway Remodeling in a Murine Model of Chronic Asthma through TLR4/NF-*κ*B Signaling Pathway

**DOI:** 10.1155/2023/3695469

**Published:** 2023-02-15

**Authors:** Ying Zhou, Xiyu Du, Qianyu Wang, Shilin Xiao, Juan Zhi, Huibin Gao, Dong Yang

**Affiliations:** Department of Anesthesiology, Plastic Surgery Hospital, Chinese Academy of Medical Sciences and Peking Union Medical College, Shijingshan District, Beijing, China 100144

## Abstract

Asthma is a common respiratory disease characterized by chronic airway inflammation. Dexmedetomidine (DEX), a highly selective *α*2 adrenergic receptor agonist, has been shown to participate in regulating inflammatory states and thus exert organ protective actions. However, the potential of DEX in asthma is still unknown. This study is aimed at investigating the role of DEX in a mouse model of house dust mite- (HDM-) induced asthma and exploring its underlying mechanism. Here, we found that DEX treatment significantly ameliorated airway hyperresponsiveness, airway inflammation, and airway remodeling in the asthmatic mice, which were similar to the efficacy of the reference anti-inflammatory drug dexamethasone. In addition, DEX reversed the increased expression of toll-like receptor 4 (TLR4) and its downstream signaling adaptor molecule nuclear factor-*κ*B (NF-*κ*B) in the lung tissue of asthmatic mice. Furthermore, these protective effects of DEX were abolished by yohimbine, an *α*2 adrenergic receptor antagonist. These results indicate that DEX is capable of ameliorating airway inflammation and remodeling in asthmatic mice, and this protective effect is associated with the inhibition of the TLR4/NF-*κ*B signaling pathway.

## 1. Introduction

Asthma is a chronic respiratory disease characterized by airway inflammation, airway hyperresponsiveness (AHR), and airway remodeling [1], with reports documenting an increasing prevalence and healthy burdens [2–4]. In some unexpected conditions, *e.g.*, during the perioperative period, an asthma attack can be easily induced by tracheal intubation or surgical manipulation, and when the acute bronchospasm is too excessive that patients are unable to ventilate and exchange gases normally, it may cause severe hypoxia or even cardiopulmonary arrest [5]. Until now, the current mainstay of asthma treatments still focuses on repressing respiratory inflammation and relieving bronchial spasms. Inhaled corticosteroids have been the most widely used anti-inflammatory agents in asthma. Nevertheless, their efficacy may be limited by intrinsic or acquired resistance [6], and long-term use of corticosteroids is associated with adverse effects, such as bone comorbidities and venous thromboembolism [7, 8]. Thus, identifying the precise molecular mechanisms of asthma that can be targeted therapeutically is in urgent requirement.

It is now well recognized that airway epithelium not only acts as a physical barrier but also orchestrates with the immune system to external disturbances [9]. The inhaled allergens, such as dust mites, pollen, or animal dander, can be sensed directly by airway epithelium via pattern recognition receptors (PRRs). Activated airway epithelial cells secrete various chemokines, cytokines, and other mediators to activate and recruit immune cells in further [10]. In this progress, Toll-like receptors (TLRs), a subgroup of PRRs, along with the downstream target, nuclear factor *κ*B (NF-*κ*B), are considered significant mediators of innate and adaptive immune responses in allergic airway inflammation [11, 12]. Particularly, TLR4, one of the type I transmembrane TLRs, has been proven to play a pivotal role in the inflammatory responses in asthma [13–15]. Therefore, the TLR4/NF-*κ*B signaling pathway may act as a potent target for developing novel treatments against asthma.

Dexmedetomidine (DEX) is a highly selective *α*2 adrenergic receptor agonist that is widely used in the intensive care units and operating rooms [16]. In addition to the sedative and hypnotic properties, accumulating evidence reports that DEX has protective effects for the brain [17], heart [18, 19], kidney [20, 21], and lung [22–24]. However, it is unclear whether DEX can also protect against asthma. In the present study, we tested the actions of DEX in a house dust mite- (HDM-) induced mouse model of chronic asthma. After treatment with DEX, lung inflammation and airway remodeling of mice were attenuated, and these changes were associated with the inhibition of the TLR4/NF-*κ*B signaling pathway. These observations provide evidence for the therapeutic potential of DEX in asthma and the underlying mechanism of its protective effects.

## 2. Materials and Methods

### 2.1. Animals

Female BALB/c mice, 5–7 weeks old, weighing 20-24 g, were purchased from Vital River Laboratory Animal Technology Co., Ltd. (Beijing, China). All animals were housed under pathogen-free conditions and a 12 : 12 hours light/dark cycle and acclimatized for 1 week before the experiments began. All the experiments were approved by the Animal Ethics Committee of Plastic Surgery Hospital, Chinese Academy of Medical Sciences and Peking Union Medical College and performed in accordance with the National Institutes of Health Guidelines for the Care and Use of Laboratory Animals.

### 2.2. Experimental Protocol

**Figure 1:**
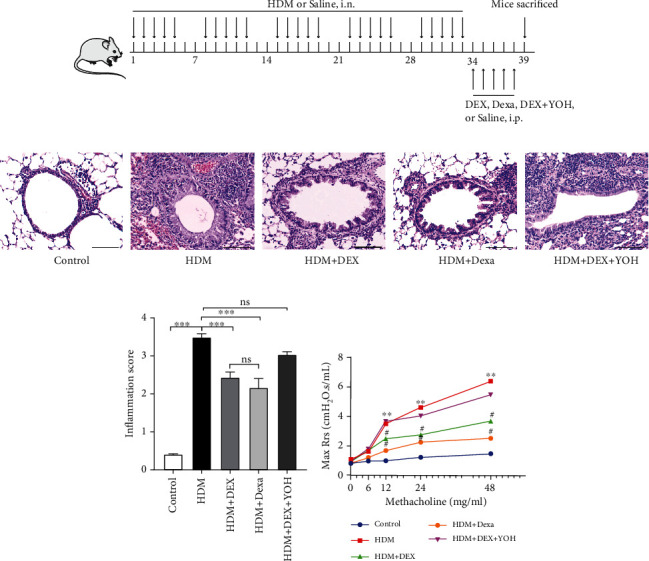
Administration of DEX attenuated inflammatory infiltration in lungs and AHR in HDM-challenged mice. (a) Schematic illustration of the protocol for chronic asthma model establishment. (b) Representative HE-stained images of lung tissue from each group (scale bar = 100 *μ*m). (c) Inflammation score estimated from the HE staining. (d) Changes of lung resistance in response to increasing doses of methacholine were evaluated 24 hours after the final treatment in each group. Data were presented as mean ± SEM (*n* = 5~8 animals). ^∗^*P* < 0.05, ^∗∗^*P* < 0.01, and ^∗∗∗^*P* < 0.001; ^#^*P* < 0.05, compared with the HDM group.

Mice were randomly assigned into the control group, HDM group, HDM+Dexa group, HDM+DEX group, and HDM+DEX+YOH group. The chronic asthma model was established according to a previously published protocol [25] with slight modifications. Briefly, mice were anesthetized using sevoflurane and then challenged with HDM extracts (Sigma, US) by intranasal instillation of 25 *μ*l HDM (1 mg/ml) for five consecutive days per week and for five consecutive weeks. Mice in the control group received 25 *μ*l saline on the same schedule. At 24 hours after the completion of the allergen challenge, mice in the HDM+Dexa group were injected with dexamethasone (Dexa) (5 mg/kg) intraperitoneally for 5 consecutive days, while mice in the HDM+DEX group were injected with DEX (25 *μ*g/kg) intraperitoneally. Specifically, to further validate the effects of DEX, yohimbine, a selective antagonist of the *α*2-adrenergic receptor, was injected intraperitoneally (1 mg/kg) at 1 hour before DEX administration in the HDM+DEX+YOH group. Mice in the control group and HDM group were injected with saline at the same timepoints. Mice were sacrificed 24 hours then for assessment of airway response to methacholine, histology, ELISA, qPCR, and western blot. The experimental protocol is shown in Figure 1(a).

### 2.3. Measurement of Lung Function and Methacholine Responsiveness

AHR was measured by the Flexivent instrument (Scireq Inc., Montreal, Quebec, Canada) 24 hours after the final drug delivery was accomplished. Briefly, mice were anesthetized with pentobarbital (10 mg/kg, i.p.). A longitudinal midline incision was made to the neck to expose the trachea, and the trachea was cannulated and connected to the Flexivent system to ventilate at 160 breaths/min, 200 *μ*l tidal. After the baseline of respiratory mechanics was recorded, mice were challenged with increasing doses of aerosolized methacholine (6, 12, 24, and 48 *μ*g/g). Total respiratory resistance (Rrs) was recorded to assess the airway hyperresponsiveness.

### 2.4. Inflammatory Cell Counts in Bronchoalveolar Lavage Fluid

Mice were performed with tracheostomy under anesthetized with pentobarbital (10 mg/kg, i.p.), and a 21-gauge lavage tube was inserted into the trachea. 0.5 ml ice-cold DPBS was slowly infused via the lavage tube into the lungs and withdrawn for 3 times to collect the BALF. The BALF was resuspended with red blood cell lysis buffer (Solarbio, China) and centrifuged at 1500 rpm for 10 min at 4°C. The supernatant was collected and stored at -80°C for further analysis, and the cell pellets were resuspended with DPBS (500 *μ*l). The total inflammatory cell number of BALF was counted by Automated Cell Counter (Thermo Fisher, US). Then, about 1-3 × 10^4^ cells were spun down to a glass slide and stained with Wright-Giemsa reagent (Solarbio, China) for differential cell counting.

### 2.5. Histopathology Analysis of Lungs

Mice were performed with thoracotomy under anesthetized with pentobarbital (10 mg/kg, i.p.), and the lungs were harvested and fixed in 4% paraformaldehyde (PFA) overnight at 4°C. To prepare paraffin-embedded tissue, the lungs were dehydrated with ethanol and then embedded in paraffin. Five-millimeter sections were cut from the paraffin blocks and stained with hematoxylin and eosin (H&E), periodic acid Schiff (PAS), and Masson trichrome. Images were observed and captured with Nikon SMZ1500 inverted microscope (Nikon).

According to the previous studies [26], the severity of lung inflammation was measured by the inflammation score based on a 5-point scoring system as follows: 0 = no inflammatory cells were observed, 1 = few inflammatory cells were observed, 2 = bronchi or vessels were surrounded by 1 layer of inflammatory cells, 3 = bronchi or vessels were surrounded by 2-4 layer of inflammatory cells, and 4 = bronchi or vessels were surrounded by more than 4 layers of inflammatory cells. The quantification of goblet cell hyperplasia in the bronchi and bronchioles was represented with a 5-point scoring system: 0 ≤ 0.5% PAS-positive cells, 1: <25%, 2: 25-50%, 3: 50-75%, and 4: >75%. The collagen deposition in the lungs was quantified by the percentage of the area occupied by collagen (blue) of the total area examined.

### 2.6. Immunofluorescence Analysis of Lungs

For the immunofluorescence staining experiment, the lungs were fixed in 4% PFA overnight at 4°C, then immersed in 30% sucrose for 3 days, and embedded in OCT. Seven-millimeter sections were cut from the OCT-embedded tissues. After permeabilization and blocking were done, lung sections were incubated with anti-*α*-SMA primary antibody (1 : 300, BOSTER, China) overnight at 4°C. The tissue slices were washed with PBS and then incubated with DyLight 488-labeled IgG secondary antibody (1 : 300, BOSTER, China) and DAPI for visualization. The positive staining area of *α*-SMA was normalized to airway basement membrane length (*μ*m) in each airway for analysis.

### 2.7. Western Blot Analysis

Lung tissues were homogenized with RIPA buffer (Applygen, China) containing a protease inhibitor cocktail (Beyotime, China). Protein concentration was determined by the BCA Protein Assay Kit (Beyotime, China), and then, samples were loaded into SDS-PAGE gels. After electrophoresis at 100 V for 90 min, the gels were transferred to polyvinylidene difluoride (PVDF) membranes (Millipore, US) for wet electric transfer at 220 mA for 90 min. After being blocked with 5% skim milk for 1 h to block the nonspecific sites, the PVDF membranes were then incubated in primary antibodies overnight at 4°C. Primary antibodies used for western blot included anti-TLR4 (1 : 1000, 66351-1-Ig, Proteintech), anti-NF-*κ*B p65 (1 : 1000, 66535-1-Ig, Proteintech), anti-phospho-NF-*κ*B p65 (p-NF-*κ*B p65, 1 : 1000, #3033, Cell Signaling Technology), anti-phospho-I*κ*B*α* (phospho-I*κ*B*α*, 1 : 1000, #9246, Cell Signaling Technology), and anti-I*κ*B*α* (1 : 1000, #4812, Cell Signaling Technology). Anti-mouse or anti-rabbit horseradish peroxidase-conjugated-IgG secondary antibody (Proteintech, China) was used to detect the binding of primary antibodies and imaged with ECL reagent (Beyotime, China) using the iBright CL1000 imaging system (Thermo Fisher Scientific). The densitometric analysis of protein bands was performed by ImageJ software, and the results of changes in protein expression levels were presented as the relative ratio of the target protein to the reference protein.

### 2.8. Real-Time Quantitative PCR

Total RNA was extracted from lung tissues using the TRIzol reagent (Thermo Fisher, US) according to the manufacturer's instructions, and the concentration of total RNA was measured by NanoDrop® 2000 ultraviolet spectrophotometer (Thermo Fisher Scientific, US). RNA was reverse-transcribed into cDNA with the TransScript® First-Strand cDNA Synthesis SuperMix kit (Beijing Transgen Biotech Co., Ltd.), and then, the quantitative PCR was performed with the LightCycler® 480 SYBR Green I Master mix kit (Roche Life Science, Swiss) according to the manufacturer's protocol. The qPCR primer sequences were designed as follows: IL-5, forward 5′-AGAATCAAACT GTCCGTGGGG-3′ and reverse 5′-TCCTCGCCACACTTCTCTTTT -3′; IL-13, forward 5′-CTCTTGCTTGCCTTGGTGGTC-3′ and reverse 5′-TGTGATGTTGCTCA GCTCCTC-3′; and *β*-actin, forward 5′-CTCTTTTCCAGCCTTCCTTCTT-3′ and reverse 5′-AGGTCTTTACGGATGTCAACGT-3′. Cycle threshold values of IL-5 and IL-13 were normalized to reference gene *β*-actin, and the 2^−*ΔΔ*Ct^ method was used to analyze the expression of transcription factors.

### 2.9. Statistical Analysis

Quantitative data are expressed as means ± standard error of mean (SEM), or as median ± interquartile range. The statistical analyses were performed using SPSS 27.0 software or GraphPad Prism 9.0. For normal distribution data, statistical significance between groups was assessed using one-way analysis of variance, followed by Bonferroni's post hoc test. Ordinal data or those with heteroscedasticity was analyzed using Kruskal-Wallis followed by Dunn's multiple comparisons test. A *P* < 0.05 was considered statistically significant.

## 3. Results

### 3.1. Administration of Dexmedetomidine Alleviates Allergic Airway Inflammation and AHR Induced by HDM

To test the therapeutic properties of DEX on chronic asthma, we first examined the effect of DEX on allergic airway inflammation and compared its efficacy with dexamethasone, the mainstay of asthma treatment. As shown in HE staining, repeated exposure to HDM caused a marked airway inflammation, characterized by abundant infiltrations of peribronchial and perivascular inflammatory cells (Figure 1(b)). Treatment with DEX significantly reduced the inflammatory infiltrates around the airway lumen and nearby vessels, which reached a similar effect as the reference anti-inflammatory drug dexamethasone (Figure 1(b)). Furthermore, pretreatment with yohimbine, a common antagonist that blocks the excitation of *α*2 adrenergic receptors, reversed the anti-inflammatory property of DEX (Figure 1(b)). Accordingly, the inflammation scores in these groups showed the same trend as that of HE staining (Figure 1(c)). These data demonstrate that allergic airway inflammation is successfully established in our chronic asthma model, and DEX is capable to alleviate chronic inflammation via *α*2 adrenergic receptors.

Since AHR is another of the hallmarks of asthma, the impact of DEX on AHR was determined additionally in the present study. Mice subjected to intranasal HDM for 5 consecutive weeks showed markedly increased maximal respiratory resistance (Max (Rrs)) to aerosolized methacholine as compared to the controls, indicating the higher reactivity and sensitivity of the asthmatic airway (Figure 1(d)). DEX administration significantly blunted the AHR under the same concentration of methacholine in mice exposed to HDM, which was in line with the results of dexamethasone. Nonetheless, pretreatment with yohimbine eliminated the inhibitory effect of DEX on AHR (Figure 1(d)). These observations further support that systemic treatment with DEX is beneficial in a model of chronic asthma.

### 3.2. Dexmedetomidine Administration Reduces the Expression of Inflammatory Cytokines in the Lungs and Inhibits Inflammatory Cell Infiltration in BALF

**Figure 2:**
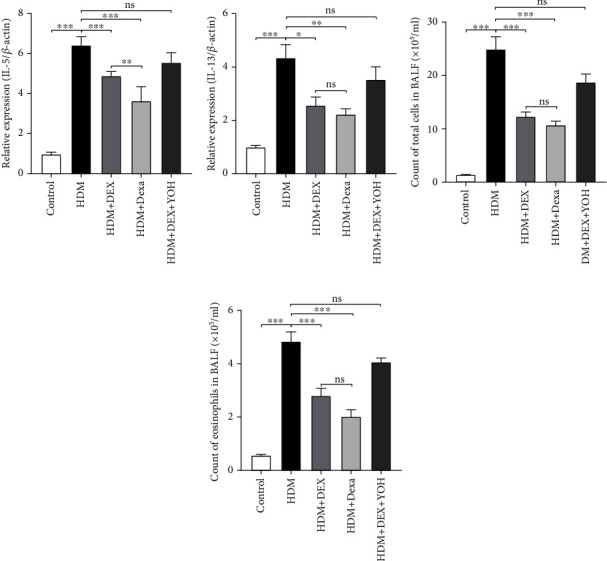
DEX treatment reduced the expression levels of inflammatory cytokines in lungs and inhibited inflammatory cell infiltration in BALF. (a, b) Cytokine gene expression assayed by RT-qPCR in lungs. (c, d) Comparison of total cell numbers and eosinophils in BALF. Data were presented as mean ± SEM (*n* = 5~8 animals). ^∗^*P* < 0.05, ^∗∗^*P* < 0.01, and ^∗∗∗^*P* < 0.001.

Since allergic asthma is dominantly accompanied by high production of type 2 cytokines [9], the expression levels of interleukin- (IL-) 5 and IL-13 in lung tissues were examined by qPCR. Mice exposed to HDM exhibited higher levels of IL-5 and IL-13 compared to the controls (Figure 2(a)). Conversely, administration of DEX restored the increased levels of IL-5 and IL-13 induced by HDM, similar to dexamethasone (Figures 2(a) and 2(b)). Remarkably, yohimbine administration reversed the effect of DEX on IL-5 and IL-13 (Figures 2(a) and 2(b)).

The anti-inflammatory effect of DEX for asthma was further validated by analyzing the BALF. As expected, the total cell number in BALF from the HDM-induced asthmatic mice was significantly higher than that in the control group (Figure 2(c)). Also, increased numbers of eosinophils were observed in the HDM group compared to the controls (Figure 2(d)). Similar to the effect of dexamethasone, DEX administration significantly decreased the total cell counts and eosinophil accumulation in BALF and was reversed by the specific inhibition of yohimbine (Figure 2(d)).

### 3.3. Dexmedetomidine Administration Inhibits Airway Remodeling Induced by HDM

**Figure 3:**
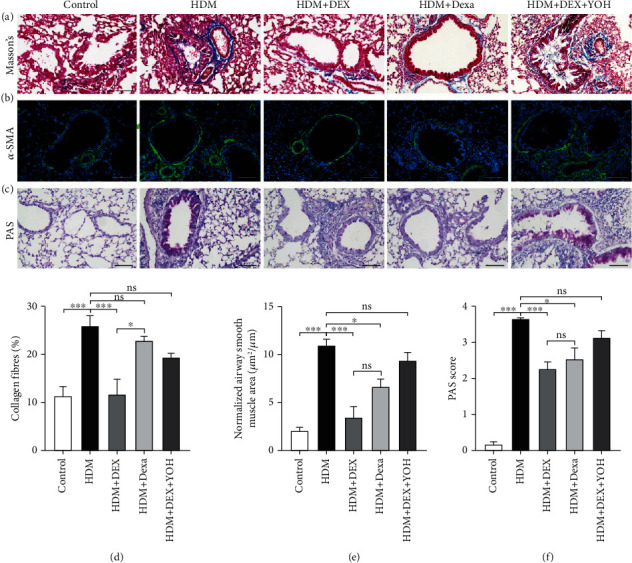
DEX administration inhibited airway remodeling in chronic asthmatic mice. Representative images of lung sections stained with Masson trichrome (a), anti-*α*-SMA antibody (green) (b), and PAS (c) from each group. Scale bar = 100 *μ*m. (d) Quantitative analyses of the percentage of collagen fiber content in lung sections. (e) Quantitative analyses of positive *α*-SMA staining area, normalized to the perimeter of the basement membrane. (f) PAS score was calculated by the percentage of epithelium positive for PAS staining. Data were presented as mean ± SEM (*n* = 5~8 animals). ^∗^*P* < 0.05, ^∗∗^*P* < 0.01, and ^∗∗∗^*P* < 0.001.

Airway remodeling refers to the structural changes both in the large and small airways, which is considered the most important factor that leads to the irreversible loss of lung function in asthmatic patients [27, 28]. Thus, the influence of DEX on goblet cell hyperplasia, peribronchial collagen deposition, and airway smooth muscle hyperplasia was evaluated. HDM-exposed mice exhibited prominent and increased peribronchial collagen deposition than those in the vehicle-treated group, as shown by Masson's trichrome staining (Figures 3(a) and 3(d)). Also, the apparently increased mass of airway smooth muscle was validated by immunostaining of *α*-smooth muscle actin (*α*-SMA) (Figures 3(b) and 3(e)). Moreover, the PAS-positive area in the lung sections of HDM-exposed mice was significantly increased than that in the controls (Figures 3(c) and 3(f)). Treatment with DEX effectively ameliorated those structural changes (Figures 3(a)–3(f)), while preventive intervention by yohimbine inhibited the antifibrotic effect of DEX (Figures 3(a)–3(f)). Of note, although not statistically significant, DEX tended to be more effective than dexamethasone in mitigating airway remodeling (Figures 3(d)–3(f)). Collectively, these results indicate that DEX has a beneficial effect on airway remodeling induced by HDM.

### 3.4. Dexmedetomidine Administration Inhibited TLR4/NF-*κ*B Signaling Pathway

**Figure 4:**
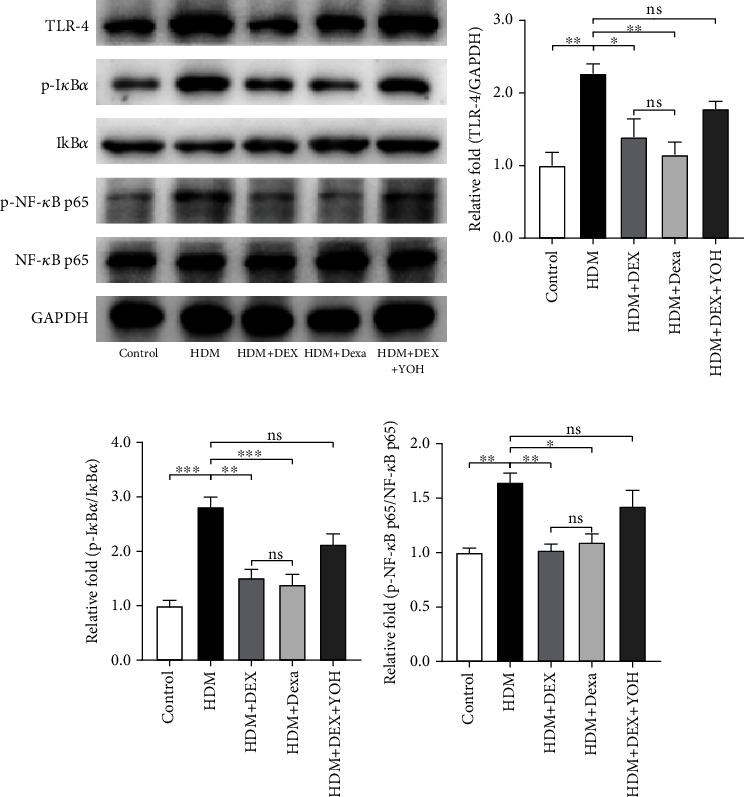
Effect of DEX on the expression levels of the TLR4/NF-*κ*B signaling pathway in lung tissues. (a) Representative images of western blots of TLR4, phosphorylated- (p-) I*κ*B*α*, I*κ*B*α*, p-NF-*κ*B p65, and NF-*κ*B p65. (b–d) Densitometric analyses of TLR4 and the ratios of p-I*κ*B*α*/I*κ*B*α* and p-NF-*κ*B p65/NF-*κ*B p65 in lung tissues form each group. Data were presented as mean ± SEM (*n* = 3~5 animals). ^∗^*P* < 0.05, ^∗∗^*P* < 0.01, and ^∗∗∗^*P* < 0.001.

To further elucidate the potential mechanism of the protective effects of DEX in asthma, we explored the activation level of the TLR4/NF-*κ*B signaling pathway in lung tissues. The western blotting results displayed that the expression of TLR4 in the lungs was markedly increased in the HDM group when compared with the controls (Figures 4(a) and 4(b)). Remarkably, both the DEX and dexamethasone prevented the elevation in the TLR4 protein content (Figures 4(a) and 4(b)). Conversely, the administration of yohimbine combined with DEX had a rare effect on the increased expression level of TLR4 induced by HDM exposure (Figures 4(a) and 4(b)).

In an unstimulated state, NF-*κ*B is sequestered by I*κ*B*α* within the cytoplasm. Upon activation, I*κ*B*α* is phosphorylated and leads to the dissociation and phosphorylation of NF-*κ*B [29]. Thus, we examined the expression levels of I*κ*B*α* and NF-*κ*B p65, as well as their corresponding phosphorylation forms in the present study. Mice exposed to HDM exhibited higher levels of phosphorylated I*κ*B*α* and NF-*κ*B p65 when compared with the saline-exposed ones (Figures 4(a), 4(c), and 4(d)). DEX as well as dexamethasone treatment after HDM challenges restored the abnormally elevated levels of phosphorylated I*κ*B*α* and NF-*κ*B p65. The effect of DEX on the activation of NF-*κ*B could be eliminated by the yohimbine (Figures 4(a), 4(c), and 4(d)). Collectively, these data further support the notion that inhibition of the TLR4/NF-*κ*B signaling pathway by DEX has a protective role in the chronic asthma model.

## 4. Discussion

In the present study, we demonstrated the protective properties of DEX and its potential mechanism in a chronic allergic asthma model. Our results revealed that DEX treatment significantly suppressed AHR, chronic airway inflammation, and airway remodeling by inhibiting the activation of the TLR4/NF-*κ*B signaling pathway. In summary, our study identified the anti-inflammatory and antifibrotic potential of DEX and provides a novel strategy for asthma.

In recent years, evidence has emerged that DEX, a widely used anesthesia adjunct, exhibits numerous anti-inflammatory mechanisms with researches reporting benefits in multiple organ protection [30, 31]. For example, DEX has been verified with potent protections in several cases of lung injury, such as ventilation-associated lung injury [24], ischemia/reperfusion injury [22], and endotoxin-induced acute injury [23, 32]. In addition, our previous study has demonstrated that DEX was capable to attenuate airway inflammation in an ovalbumin-induced asthma model [33]. However, these studies mainly focused on the efficacy of DEX in the acute phase of diseases, ignoring that the asthmatic respiratory tract is in a state of chronic inflammation. To establish a more clinical relevant model to test the protective effects of DEX in chronic asthma, we employed HDM, the most important aeroallergen for allergic response in clinical [34], and adopted a repeated exposure procedure in the present study.

AHR is defined by the increased sensitivity and reactivity of airways in response to both specific and nonspecific stimuli [35], which represents the functional changes in asthma that contribute to airway obstruction. The presence of AHR is of important clinical significance not only for diagnosing asthma but also for predicting the risks for the decline in lung function [36, 37]. Our results demonstrated that treatment with DEX in the murine model of chronic asthma substantially reduced AHR to the normal level, similar to earlier reports [33, 38, 39]. DEX has a favorable profile in sympatholysis and drying of respiratory secretions; thus, it benefits the suppression of airway reflexes in awake intubation and anesthesia emergencies [40]. However, the understanding for the exact mechanisms of DEX in AHR is limited. It has been recognized that AHR has two components, one of which is persistent and associated with airway remodeling and chronic airway inflammation, and the other is variable that can be induced by episodic exposure to allergens [35]. Although DEX is suggested to attenuate acetylcholine release and C-fiber-mediated airway smooth muscle contraction [41], the evidence of its immediate effect on airway response is still insufficient. Since we adopted a chronic asthma model here, the phenotypic features of airway inflammation and airway remodeling were further examined in asthmatic mice after DEX treatment to understand the possible underlying mechanism.

Initially, our histological results showed a protective role of DEX in inflammatory cell infiltration, epithelial cell damage, and airway wall thickening, which are consistent with our previous findings using an acute asthma model [33]. Airway inflammation orchestrated by type 2 helper T cell (Th2) response has been considered the key feature of asthma [9]; therefore, we focused on the modulatory role of DEX in Th2 immunity. In the present study, we observed that DEX efficiently alleviated the infiltration of eosinophils and neutrophils in BALF and decreased the production of IL-5 and IL-13, two classic Th2 cytokines. These findings were partly aligned with researches in other disease models, in which DEX inhibited the inflammatory reactions via targeting various cytokine pathways [42, 43]. Intriguingly, clinical evidence indicates that DEX is capable to shift the Th1/Th2 balance toward Th1 in perioperative stress and thus protects the immune function [44–46]. As mentioned above, the Th2 response is the main driver of asthma. Treatments that mediated the Th1/Th2 ratio were verified to ameliorate asthmatic airway inflammation [47–49]. Altogether, these findings suggest the strong potential of DEX in protecting against allergic airway inflammation, and there may be a common mechanism implicated in the anti-inflammatory activity of DEX in different diseases.

Airway remodeling is another pathological hallmark of chronic asthma. In our *in vivo* experiment, the pathological changes in the asthmatic airway, including goblet cell hyperplasia, extracellular matrix deposition, and increased airway smooth muscle mass were mitigated by DEX treatment. The suppressive effect of DEX on fibrosis has been investigated in various tissues and organs [50–52]. For example, in an acute kidney injury model, pretreatment with DEX alleviated tubulointerstitial fibrosis and the expression of inflammatory markers via the inhibition of *α*2-adrenergic receptor and hence hindered the progress of chronic kidney disease [51]. In most cases, these structural changes are attributed to a persistent inflammatory state of the airway [28]. Based on the protective effect of DEX on airway inflammation we discussed earlier, the impact of DEX on airway remodeling in the present study is supposed to be the consequence of its anti-inflammatory effects. Of note, there are conflicting data about the role of DEX in tissue fibrosis. Activation of the *α*2 adrenergic receptor by DEX enhanced the proliferation and differentiation of fibroblasts, resulting in the increased deposition of extracellular matrix [53, 54]. Additionally, airway remodeling can be developed independently in asthma [55, 56], which may be associated with the direct participation of airway smooth muscle hypertrophy or neural mechanism in the disease progression [27]. Owing to the controversial findings of DEX in fibrosis, the use of DEX as a modulator in asthmatic airway remodeling warrants further investigations.

Except for immune cells, airway epithelium is capable to recognize pathogens directly by PRRs expressed on them and sequentially activates the innate immune system [57]. In the allergic inflammation setting, the most well-studied type of PRRs is the TLR family. Both clinical and preclinical evidence reveals that TLR4 implicates in the development of allergen-induced Th2 responses [13]. Particularly, Derp 2, a major component of HDM, has been proven to stimulate and augment the TLR4 pathway in asthma [58]. TLR4 signals trigger the downstream signaling cascade, leading to the activating of NF-*κ*B, which induces the release of proinflammatory cytokines and chemokines in further [59]. To confirm the possible mechanism of DEX in protecting against chronic asthma, we explored the activation of the TLR4/NF-*κ*B signaling pathway after DEX intervention. Our results illustrated that DEX significantly decreased the systematic expression levels of TLR4 and restrained the activation of NF-*κ*B signaling pathway in lung tissue. These findings agreed with other researches which declared that DEX had inhibitory effects on the expression of TLR4 and the downstream effector NF-*κ*B [60, 61]. The changes observed in this signaling pathway were coincident with the suppression of pathological changes in the airway after DEX treatment. Also, our group previously reported that TAK-242, a specific TLR4 signaling inhibitor, had similar effects with DEX on airway inflammation in asthmatic mice [33]. Therefore, it is conceivable that DEX protected against allergic airway inflammation by inhibiting TLR4/NF-*κ*B signaling.

Our study has several limitations. Firstly, *α*2 adrenergic receptors are expressed on both immune and nonimmune cells [62]. In the present study, we only observed the outcomes of the lung after DEX intervention but did not explore how DEX affects the function of various immune cells. A detailed mechanistic study to clarify the respective effects of DEX on airway structural cells and immune cells in asthma is needed in further researches. Secondly, internalizing disorders, such as anxiety and depression, have been found to be closely connected with chronic airway inflammation from asthma [63]. Since DEX has potent sedative and anxiolytic properties, the influence of DEX on internalizing conditions using behavior tests should be assessed to fully understand the potential mechanisms of DEX's therapeutic effects that are implicated in asthma.

## 5. Conclusion

In conclusion, the present research has shown a potential benefit of DEX that modifies both airway inflammation and airway remodeling in a chronic asthma model through the inhibition of the TLR4/NF-*κ*B activity, which provides a novel therapeutic strategy for asthma.

## Data Availability

All data generated or analyzed during this study are included in this article.
